# Whether do plant cells sense nitrate changes without a sensor?

**DOI:** 10.3389/fpls.2022.1083594

**Published:** 2022-11-24

**Authors:** Yu-Fan Fu, Lin-Bei Xie, Xin-Yue Yang, Zhong-Wei Zhang, Shu Yuan

**Affiliations:** College of Resources, Sichuan Agricultural University, Chengdu, China

**Keywords:** nitrate transporter NRT1.1, transcription factor NLP7, nitrate sensor, nitrate signaling, adenosine monophosphate-activated protein kinase

## NRT1.1-dependent and NRT1.1-independent nitrate signaling pathways

Nitrogen is a key nutrient macroelement. Nitrate is the most abundant inorganic form of N in soils for plant absorption, and works as a signaling molecule that regulates multiple growth and developmental processes (Fredes et al., 2019; Vidal et al., 2020; Li et al., 2021), such as root elongation (Zhang et al., 2019), leaf expansion (Yang et al., 2022) and flowering (Yuan et al., 2016; Zhang et al., 2021). However, nitrate signaling in plant cells remains largely unknown. Both a proton-coupled transporter NRT1.1 (CHL1; Ho et al., 2009) and the NIN-like protein (NLP) transcription factor NLP7 (Liu et al., 2022) have been suggested as nitrate sensors.

The nitrate transporter NRT1.1 is a dual-affinity nitrate transceptor controlling the primary nitrate response (nitrate signaling), in which expressions of nitrate assimilation genes and nitrate transporter genes are induced rapidly by nitrate treatments (Ho et al., 2009). NRT1.1 facilitates not only nitrate uptake but also auxin transport. Nitrate treatments repress NRT1.1-mediated auxin uptake, indicating that the nitrate signaling *via* NRT1.1 is correlated with a regulation of auxin transport (Krouk et al., 2010). The T101 residue of NRT1.1 could be phosphorylated by calcineurin B-like interacting protein kinase 23 (CIPK23; Ho et al., 2009). The phosphorylation state of NRT1.1 plays an important role in regulating lateral root development by modulating nitrate-mediated basipetal auxin export and nitrate-dependent signal transduction (Zhang et al., 2019). Thus, NRT1.1 is a master switch that integrates nitrate signaling/transport and auxin signaling/transport. However, mutation of NRT1.1 promotes both lateral root growth and auxin accumulation in these roots at low nitrate levels, but not at high levels (Krouk et al., 2010) And the null *chl1-5* mutant resembled wild-type plants when grown on medium with nitrate as the sole nitrogen source (Liu et al., 2022). The activation of primary nitrate response genes were only partially repressed in *chl1-5* mutant (Liu et al., 2022). Thus, there should be NRT1.1-independent nitrate signaling pathways.

## NLP7 is insensitive to nitrate changes, when the cytosol nitrate concentrations are higher than 1 mM

The NIN-like protein (NLP) transcription factor NLP7 functions as a master switch, which controls the expression of a large number of genes in response to nitrate changes (Liu et al., 2017). Recently, Liu et al. (2022) indicated that nitrate directly binds to NLP7, and NLP7 is derepressed upon nitrate perception *via* its N terminus. Transcriptome reprogramming in primary nitrate responses triggered by nitrate was abolished in *nlp7* mutant (Liu et al., 2022). However, none of the previously-reported receptors have been identified as transcription factors so far. For all known receptors (sensors), downstream of binding with signaling molecules, there are usually multiple elements involved in the signaling pathway, such as mitogen-activated protein kinase (MAPK) cascade (Liu, 2012). If nitrate acts on NLP7 directly, there will be no crosstalk with other signals and no possibility of positive or negative feedback regulations, which does not conform to the evolutionary law.

The dissociation constant K_d_ value of NLP7 binding to nitrate was about 50 μM (Liu et al., 2022). However the K_d_ values of phytohormone receptors range from 4 nM to 50 nM, which are about 1000 times lower than that of NLP7 (**Table 1**). Even for the nonspecific amino-acid receptors, glutamate receptor-like (GLR) channels, their K_d_ value can be as low as 0.33–5.5 µM (Alfieri et al., 2020), which are still 9–150 times lowers than that of NLP7 (**Table 1**). Therefore, NLP7 is not a specific nitrate receptor.

**Table 1:** Dissociation constant (K_d_) values of phytohormone, amino-acid receptors (sensors) and nitrate transporters.

Signaling molecule	Receptor (Sensor)	K_d_	Reference
Auxin (Indole-3-acetic acid; IAA)	Transport Inhibitor Response 1 (TIR1)	18 nM	Calderón Villalobos et al., 2012
Cytokinin (CTK)	Histidine Kinases (HK)	4.0 nM to trans-zeatin	Stolz et al., 2011
Gibberellin (GA)	GA Insensitive Dwarf 1 (GID1)	30 nM to GA_4_	Nakajima et al., 2006
Abscisic acid (ABA)	Protein Phosphatases Type 2C (PP2C)	38 nM	Santiago et al., 2009
Ethylene (ET)	Ethylene Receptor 1 (ETR1)	1.24 μl/liter	McDaniel and Binder, 2012
Salicylic acid (SA)	Non-expressor of Pathogenesis Related protein 4 (NPR4)	50 nM	Wang et al., 2020
Jasmonic acid (JA)	Coronatine-Insensitive 1 (COI1)	48 nM	Sheard et al., 2010
Amino acid (AA)	Glutamate receptor-like (GLR) channels (nonspecific receptors in plants)	0.33 µM to Cys; 2.2 µM to Glu; 5.5 µM to Gly	Alfieri et al., 2020
*Synechococcus* sp. Strain PCC 7942	Nitrate transporter A (nrtA)	0.3 μM	Maeda and Omata, 1997
*Staphylococcus carnosus*	Nitrate regulatory element A (NreA)	22 μM	Niemann et al., 2014
*Arabidopsis thaliana*	Nitrate transporter NRT1.1	1 mM	Parker and Newstead, 2014

A nitrate transporter in the cyanobacterium *Synechococcus*, NrtA, was shown to bind nitrate and nitrite with a high affinity (K_d_ = 0.3 μM; Maeda and Omata, 1997). Comparatively, another nitrate transporter in *Staphylococci*, NreA, was shown to bind nitrate with a low affinity (K_d_ = 22 μM; Niemann et al., 2014; **Table 1**). Thus, NLP7 may have a function as a nonspecific transporter with low micromolar affinity. Similarly, Ethylene Insensitive2 (EIN2) contains the 12-transmembrane domain of the NRAMP family of metal transporters, but has no capacity for metal transport. Instead, EIN2 functions an essential ethylene signaling component in higher plants (Alonso et al., 1999). NLP7 shares some similarity with cyanobacterial nitrate transporters (Liu et al., 2022), however, in higher plants, it has developed a new function, serving as a transcription factor. This suggests that the role of NLP7 as a nitrate transporter might have been diminished largely from its ancient roots.

On the other hand, the contents of nitrate in plant cells are very high, even under low N conditions (North et al., 2009). For instance, when the N level in MS medium was reduced to 1/20, the whole seedling nitrate content only reduced from 10 mM to 7 mM (Fu et al., 2020; Yang et al., 2022). Another study demonstrated that, by the time the barley roots had been out of nitrate for 24 h, the cytosol nitrate contents in root epidermal cells only decreased from 4.6 mM to 4.0 mM, and the cytosol nitrate contents in root cortical cells only decreased from 3.7 mM to 2.9 mM (van der Leij et al., 1998), when the nitrate-starvation signal has been already triggered. Nitrate concentration is 1 to 5 mM in the cytosol and 5 to 75 mM in the vacuole under nitrogen sufficient conditions. Under nitrogen deficient conditions, the nitrate concentration of cytosol was maintained stable by export of nitrate from the vacuole. However, the stored nitrate in vacuoles can only lasted for two days if there is no nitrogen was provided (Cookson et al., 2005; Marschner and Rengel, 2012). Although the exact cytosol nitrate concentration under N deficient condition has not been well-documented, Arabidopsis root nitrate levels decreased from 10 mM to 1 mM, and its shoot nitrate levels decreased from 90 mM to 4 mM after 7 days of N starvation (Bussell et al., 2013). Nevertheless, for NLP7, the saturated binding can be achieved when the nitrate level is higher than 1 mM (Liu et al., 2022). In other words, even under low-N conditions, NLP7 remains in a saturated binding state, so it is unlikely to respond to nitrate changes, when the cytosol nitrate concentrations are higher than 1 mM.

NRT1.1 has also been suggested as a nitrate sensor (Ho et al., 2009). Although the K_d_ value of NRT1.1 to nitrate was very high (about 1 mM) (Parker and Newstead, 2014), in conditions of high nitrate availability (> 1 mM), NRT1.1 behaves as a low-affinity transporter (K_m_ = 4 mM); when nitrate levels fall below 1 mM, NRT1.1 is switched into a high-affinity mode (K_m_ = 40 μM; Liu et al., 1999). Compared with NLP7, NRT1.1 is more likely to be responsive to exogenous nitrate changes.

## The binding domain and the signaling domain are separated in NRT1.1, but not in NLP7

It is necessary to prove that the binding of NLP7 with nitrate is a prerequisite for its transcriptional activation function. Liu et al. (2022) showed that *4xNRE(nitrate response cis-element)-min(35S minimal promoter)-LUC(luciferase gene)* was activated by NLP7 only in the presence of nitrate in both nitrate-free Arabidopsis leaf cells and 293T human cells. However, it could not rule out a possibility that some nitrate-responsive factors conserved in both plants and animals (humans) regulates NLP7 activity upon nitrate treatments. The adenosine monophosphate-activated protein kinase (AMPK) proteins could be candidates for this process. Arabidopsis AMPKα1 homologs KIN10 and KIN11 (AT3G29160) proteins share 79.3% similarity with the human AMPKα1 (Yuan et al., 2016). KIN10 phosphorylated NLP7, and promoted its cytoplasmic localization and degradation (Wang et al., 2021a). Nitrate depletion induced KIN10 accumulation, whereas nitrate treatment promoted KIN10 degradation (Wang et al., 2021a). In both plant cells and mammalian cells, nitrate availability regulates AMPK activities positively (Yuan et al., 2016; Li et al., 2022).

Generally, the binding domain and the signaling domain of receptors are separated, and there will be a mutant receptor that cannot bind to the signaling molecules but can activate downstream factors (e.g. kinase activity), or there will be a mutant receptor that can bind to the signaling molecules but cannot conduct a downstream signal. Liu et al. (2022) did not construct such mutants. They did demonstrate that substitutions of conserved residues in the ligand-binding pocket (the HLY/AAA mutation) impaired the ability of nitrate-triggered NLP7 to control transcription, transport and plant growth. However, given that both the DNA binding domain and nitrate binding sites are at the N-terminal (Liu et al., 2022), the NLP7 (HLY/AAA) mutant protein may not be able to bind nitrate or have a transcriptional activation activity.

Contrastingly, NRT1.1 works as a nitrate sensor independent of its transporter activity. The *chl1-9* mutant with a point mutation of Pro492 to Leu, was found to have normal levels of NRT1.1 transcript and protein (Liu et al., 1999). *chl1-9* has been shown to be defective in both high- and low-affinity nitrate uptake. Interestingly, despite the nitrate uptake defect, *chl1-9* still showed normal biphasic primary nitrate responses and typical K_m_ values in both high- and low-affinity ranges (Ho et al., 2009). Consistently, when the *CHL1-9* full-length cDNA was introduced into the null mutant *chl1-5*, signaling defects but not the uptake defect could be rescued (Ho et al., 2009). These results showed that the transporter activity was not needed for the sensing function of NRT1.1. As mentioned above, NRT1.1 also mediates auxin transport and signaling (Krouk et al., 2010; Zhang et al., 2019), it would be very interesting to investigate whether *chl1-9* mutant is defective in basipetal auxin export and/or nitrate-dependent auxin signaling.

## Nitrate signaling through AMPK pathways may not require a nitrate sensor

We speculate that either the nitrate binding capacity of NLP7 is not related to its transcriptional activation activity, or that, even if it is related, the change of nitrate at high concentrations (> 1 mM, when NLP7 is saturated bound with nitrate) may be mainly perceived through other proteins. Nitrate represses ferredoxin-NADP^+^-oxidoreductase (*FNR1*) expression, thereby contributing to declines in NADPH/NADP^+^ and ATP/AMP ratios, which in turn activates AMPK (KIN10 and KIN11) and modulates its nuclear abundance (Yuan et al., 2016). KIN10 phosphorylates NLP7 to induce its cytoplasmic retention and the subsequent degradation (Wang et al., 2021a). Under the normal growth condition, nitrate activates NRT1.1-CNGC15 (cyclic nucleotide-gated channel protein 15) complex to produce ${\text{NO}}_{3}^{-}$ specific Ca^2+^ signature (Wang et al., 2021b), which results in the NLP7 phosphorylation by Ca^2+^-sensor protein kinases (CPKs) at Ser205, thereby triggering NLP7 nuclear localization, which regulates downstream gene expression and promotes plant growth (Liu et al., 2017). Nevertheless, under nitrate-deficient conditions, KIN10 is induced to phosphorylate Ser125 and Ser306 of NLP7 protein, which increases its cytoplasmic localization and the subsequent degradation, therefore repressing nitrate-regulated gene expression and inhibiting growth (Wang et al., 2021a). These pathways may not require a nitrate sensor (like NRT1.1), but may be regulated through changes in cellular nitrate levels. So far, the nitrate signaling panorama is still incomplete. The role of KIN11 and its correlation with KIN10 in nitrate signaling are still unclear. The association between CPKs and AMPK (who is the upstream kinase, who is the upstream kinase, or they could phosphorylate each other) requires further studies. And the relationship between NRT1.1 (sensor)-dependent nitrate signaling and NRT1.1-independent nitrate signaling also needs further investigations.

## Author contributions

SY conceived the project. Y-FF, L-BX, X-YY, and Z-WZ. performed the literature search. SY wrote the manuscript with input from Y-FF, L-BX, X-YY, and Z-WZ. All authors contributed to the article and approved the submitted version.

## Funding

This work was supported by the Sichuan Province Youth Science and Technology Innovation Team (20CXTD0062) to SY and the Applied Basic Research Program of Sichuan Province (2020YJ0410) to Z-WZ.

## Acknowledgments

We thank Miss. Hannah Elizabeth Levengood (Purdue University, USA) for the linguistic assistance during the preparation of this manuscript.

## Conflict of interest

The authors declare that the research was conducted in the absence of any commercial or financial relationships that could be construed as a potential conflict of interest.

## Publisher’s note

All claims expressed in this article are solely those of the authors and do not necessarily represent those of their affiliated organizations, or those of the publisher, the editors and the reviewers. Any product that may be evaluated in this article, or claim that may be made by its manufacturer, is not guaranteed or endorsed by the publisher.
